# Improving the Mechanical Properties of a Lattice Structure Composed of Struts with a Tri-Directional Elliptical Cylindrical Section via Selective Laser Melting

**DOI:** 10.3390/ma16155487

**Published:** 2023-08-06

**Authors:** Xiong Xiao, Liangwen Xie, Xianyong Zhu, Jiaan Liu, Yanru Luo, Peng Song, Jiali Zhao, Jinyuan Zhang, Chen Wang, Song Yang, Peng Wu, Xiangmi You, Cheng Jiang

**Affiliations:** 1 School of Mechanical and Aerospace Engineering, Jilin University, Changchun 130022, China; xiaoxiong20@mails.jlu.edu.cn (X.X.); xlw1126@163.com (L.X.); luoyr@jlu.edu.cn (Y.L.); songpenglq@163.com (P.S.); zhaojiali88@163.com (J.Z.); jinyuan22@mails.jlu.edu.cn (J.Z.); chenwangowen@163.com (C.W.); yangsongjlu@jlu.edu.cn (S.Y.); 2 Chongqing Research Institute, Jilin University, Chongqing 401120, China; 3 College of Materials Science and Engineering, Jilin University, Changchun 130022, China; liuja@jlu.edu.cn; 4 Changchun Baoze Technology Co., Ltd., Changchun 130051, China; bzwupeng@163.com; 5 CISDI Group Co., Ltd., Chongqing 401122, China; xiangmi.you@cisdi.com.cn

**Keywords:** lattice structure, strut, variable cross-section, finite element analysis, compression test, energy absorption

## Abstract

In recent years, lattice structures produced via additive manufacturing have been increasingly investigated for their unique mechanical properties and the flexible and diverse approaches available to design them. The design of a strut with variable cross-sections in a lattice structure is required to improve the mechanical properties. In this study, a lattice structure design method based on a strut cross-section composed of a mixture of three ellipses named a tri-directional elliptical cylindrical section (TEC) is proposed. The lattice structures were fabricated via the selective laser melting of 316L alloy. The finite element analysis results show that the TEC strut possessed the high mechanical properties of lattice structures. Compression experiments confirmed that the novel lattice structure with the TEC strut exhibited increases in the elastic modulus, compressive yield strength, and energy absorption capacity of 24.99%, 21.66%, and 20.50%, respectively, compared with the conventional lattice structure at an equal level of porosity.

## 1. Introduction

Additive manufacturing largely bridges the gap between innovative design and advanced manufacturing [1,2,3,4,5]. As a typical example of an advanced structure, additive manufacturing lattice structures are becoming increasingly important in promoting the development of lightweight, multifunctional, intelligent, and biomimetic materials and structures.

Over the past few years, lattice structures have been regularly arranged in both two-dimensional and three-dimensional spaces. They are composed of interconnected masts and node units, providing a wide range of interoperability performances and application opportunities. Table 1 lists typical lattice structures, including the cubic structure [6], quasi-crystalline structure [7], three-period minimal surface structure [6,8,9,10,11], plate-strut hybrid structure [12], pierced-plate structure [13], curved-strut structure [14], structural asymmetrical structure [14], plate structure [15], auxetic structure [16,17], of other novel structures [18].

More and more research has been conducted on lattice structures with variable cross-sections. Carlos et al. examined and measured the effect of rectangular columns on the mechanical performance of porous structures, as well as the contribution of nodes to the effective stiffness of beams [19]; Thomas et al. investigated the effect of tapered struts on the mechanical properties of porous structures and found that such porous structures possessed greater mechanical properties [20]; Qi et al. proposed a porous structure consisting of octagonal trusses of tapered beams and truncated octahedral unit cells, which significantly increased the modulus and reduced the anisotropy of the porous structures [21].

Although there are many design methods for lattice structures with variable cross-sections, there is limited information on the design of struts with 3D elliptical cross-sections. Inspired by various cross-section industrial structures, such as railroad tracks and I-beam steel frames, a special variable cross-section structure design method was applied to the porous structure to improve the mechanical properties of the porous structure. In this study, a new design method was proposed for a lattice structure with a strut consisting of a mixture of three perpendicular ellipses in the cross-section, called a triple elliptic cylindrical cross-section (TEC). The TEC, which has a “petal-like” shape, has unique feature and a beautiful shape, and can be used for targeted parametric design of lattice structures to control the shape parameters of elliptic curves, such as the long axis, short axis, and eccentricity, with a greater possibility of diversified parameter control. The properties of this lattice structure with a variable cross-section were investigated by comparing the traditional BCC porous structure and the novel BCC porous structure through numerical simulation techniques. By altering the shape of the strut, the porous 316L lattice structure produced using the SLM process was experimentally validated to accomplish the desired improvement in mechanical properties.

Advanced lattice structures with higher compression resistance and energy absorption capabilities play an important role in many fields, such as the development of impact-resistant armored-vehicle door panels and tank armor plates for the protection of soldiers from weapons; aircraft shells and engine parts to prevent flight accidents due to bird impact; and submarines for high-pressure environments.

## 2. Materials and Methods

### 2.1. Structure Design

**Figure 1:**
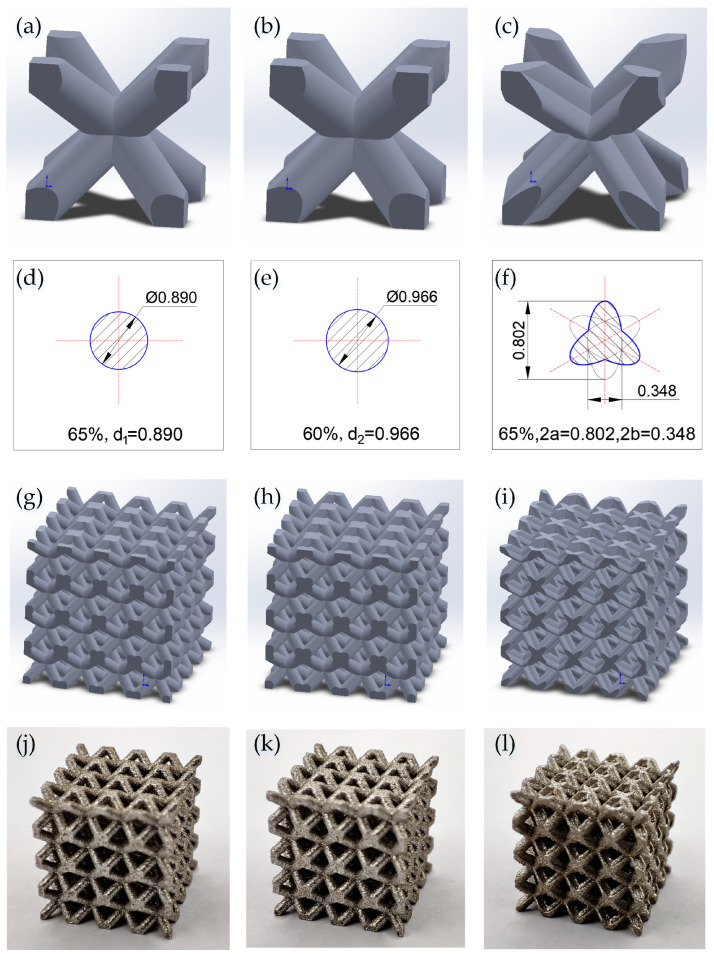
Schematic illustrations (1 × 1) of the BCC-65, BCC-60, and BCC-TEC65 are shown in (**a**–**c**), respectively. The dimensions of the three models are shown in (**d**–**f**), respectively. The geometric configurations (4 × 4) of the three models are shown in (**g**–**i**), respectively. The SLMed samples (4 × 4) of the three models are shown in (**j**–**l**), respectively.

In this paper, the cross-section of a strut consisting of a mixture of three ellipses was designed and named the tri-directional elliptical cylindrical section (TEC). The conventional body-centered cubic (BCC) structure with this strut cross-section was named the body-centered cubic with tri-directional elliptical cylindrical section (BCC-TEC), and a new structure design method was developed that improved the mechanical performance by changing the strut cross-section. The architected BCC-TEC unit cell illustrated in Figure 1c was established based on the TEC.

As shown in Figure 1a–i, in order to construct a periodic lattice structure, 3 unit cells with an identical side length of 3000 µm were designed, named BCC-65, BCC-60, and BCC-TEC65, respectively. BCC-65 and BCC-60 were body-centered cubic BCC structures with 65% and 60% porosity and strut diameters of 0.890 mm and 0.966 mm, respectively, as shown in Figure 1d,e. BCC-TEC65 was a BCC-TEC structure with 65% porosity, and the strut cross-sections were a combination of three ellipses, each with 2a = 0.802 mm, 2b = 0.348 mm, and e = 0.9, where 2a was the long axis, 2b was the short axis, and e was the eccentricity. The centers of all three ellipses coincided with the center of the strut section. The angle between the three ellipses’ central axes was 120 degrees, and the cross-section shapes were taken from the curves of the corresponding parts of the three ellipse diagrams, as shown in Figure 1f. The cross-sectional dimensions of the unit cells did not change in the direction of the axis. The models of BCC-65, BCC-60, and BCC-TEC65 with 4 × 4 × 4 cells were 12 mm in length.

### 2.2. Finite Element Analysis

**Figure 2:**
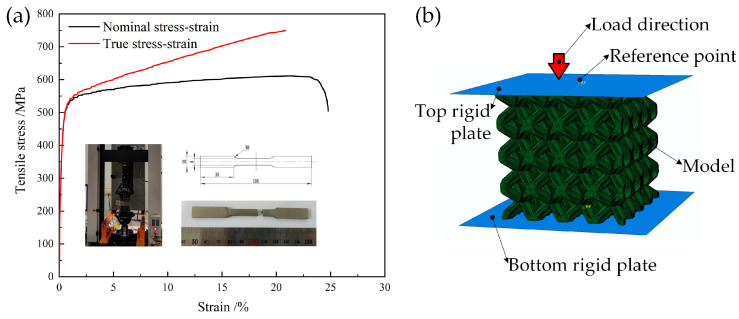
Nominal stress-strain curve and true stress-strain curve of the 316L tensile specimen (**a**); 4 × 4 model for finite element analysis (**b**).

To obtain the performance parameters of 316L in the printed state required for the numerical simulation analysis, several 316L tensile specimens in the printed state were prepared, and the tensile specimens, after printing and forming, were tested using an In-stron-1121 tensile tester with 1 mm/min collet displacement speed. To accurately measure the elastic modulus of the specimen, an electronic extensometer was clamped in the middle part of the specimen, which had a scale length of 25 mm. When the elongation exceeded 18%, the extensometer was removed until the specimen was pulled off, and then the specimen was replaced, and the experiment was repeated. The tensile specimen size and stress-strain curve are shown in Figure 2a. The nominal stress-strain curve shows that the printed state of 316L had a modulus of elasticity of 196 GPa, a nominal yield strength of 469 MPa, and a nominal tensile strength of 598 MPa.

Abaqus/Explicit was used to simulate the quasi-static deformation behavior of the 4 × 4 × 4 models under uniaxial compression. The whole compression model of the lattice structure was composed of five parts: the load direction, the reference point, the top plate, the model, and the bottom plate, as presented in Figure 2b. To simulate quasi-static compression, the bottom plate was fixed while the top plate was moved at a rate of 1 mm/min along the z-axis while all other degrees of freedom were held constant. Self-contact was set to general contact. Friction formulation of tangential behavior was set to penalty and the friction coefficient was assigned as 0.15. Ten-node modified quadratic tetrahedron components were used to mesh the models (C3D10M). Density was assigned as 7.93 × 10^−9^ t/mm^3^, and plasticity was determined according to the stress-strain curve, as shown in Figure 2a. Young’s modulus was assigned as 196 GPa according to the previous tensile stress-strain curve. Poisson’s ratio was assigned as 0.3 according to the previous research [22,23,24]. A desktop workstation with 28 CPUs ran all simulations.

### 2.3. Specimen Fabrication

**Figure 3:**
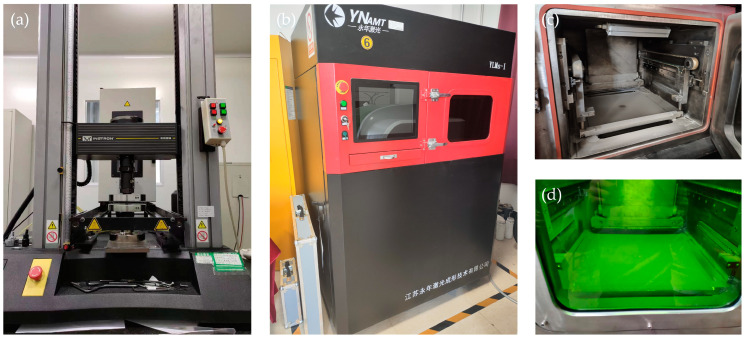
Instron-5869 universal testing machine (**a**), YLMs-1 selective laser melting machine (**b**), non-working (**c**), working (**d**).

A YLMs-1 selective laser melting machine (Jiangsu Yongnian Laser Forming Technology Co., Ltd., Kunshan, China) was used to fabricate the lattice structures in this study, as shown in Figure 3b–d. The particles of the SS316L, which ranged from 15 μm to 50 μm, were melted layer by layer with an input energy of 170 W, a laser scan speed of 900 mm/s, a hatching spacing of 70 μm, and a layer thickness of 30 μm. The chemical composition of the SS316L powder was 2.79 wt.% C, 1.47 wt.% Mn, 0.03 wt.% P, 0.02 wt.% S, 0.72 wt.% Si, 16.72 wt.% Cr, 11.92 wt.% Ni, and 2.13 wt.% Mo with balanced Fe.

The models of BCC-65, BCC-60, and BCC-TEC65 were fabricated as cubes containing 4 × 4 × 4 unit cells and strut details, as shown in Figure 1j–l. In order not to affect the experimental results, all compressed parts were cut using a wire-cutting machine and then cleaned with ultrasonic waves.

### 2.4. Forming Quality

**Figure 4:**
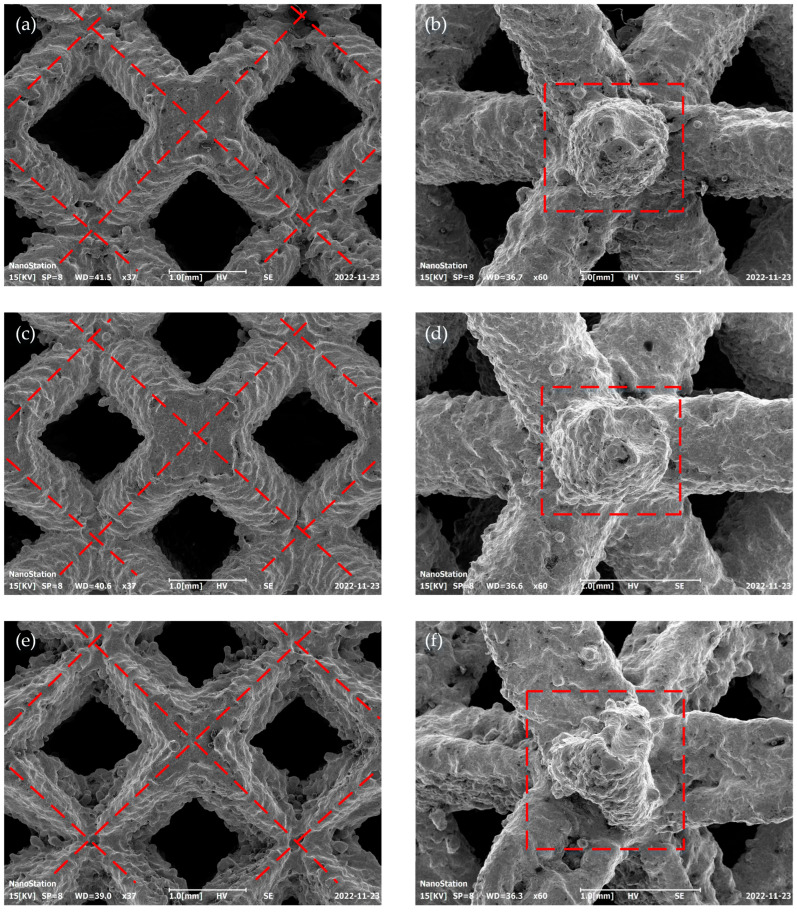
SEM morphologies (front view on the left and column cross-section on the right) of the BCC-65 (**a**,**b**), BCC-60 (**c**,**d**), and BCC-TEC65 (**e**,**f**) models.

**Figure 5:**
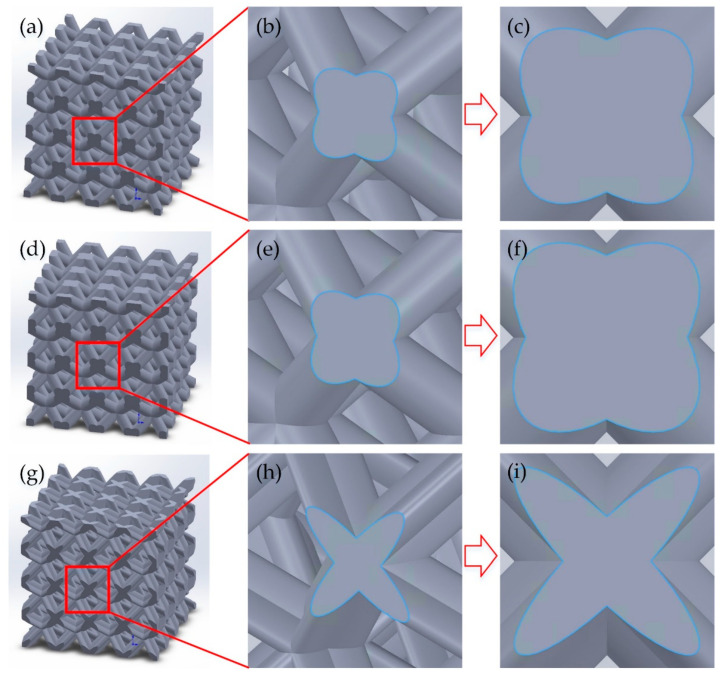
The node section shape (4 × 4 models on the left, enlarged pictures in the middle, and enlarged front view on the right) of the BCC-65 (**a**–**c**), BCC-60 (**d**–**f**), and BCC-TEC65 (**g**–**i**) models.

A scanning electron microscope (SEM, JEOL JSM-7900F, Tokyo, Japan) was used to observe the microscopic morphologies of local units of the BCC-65, BCC-60, and BCC-TEC65 structures. The front view and column cross-section of the structures are shown in Figure 4. The viewing direction of the node section shape is shown in Figure 5. The node section shape of BCC-65 and BCC-60 were the same, while the node section shape of BCC-TEC65 was similar to a 4-pointed star shape, which was beneficial for the mechanical properties. This was confirmed by the experimental results previously reported by Carlos et al. [19]. In addition to conforming to the geometric geometry of the designed models, the three structures’ details were largely complete. However, the surface roughness was slightly lacking, and the formed surface was uneven, with visible particulates and traces of laser scanning point melting.

### 2.5. Mechanical Performance Test

With loading in the z-direction, uniaxial quasi-static compression tests of the lattice structures were carried out using an Instron-5869 universal testing machine at a speed of 1 mm/min, as shown in Figure 3a. A high-speed camera was used to record the process of compression. To reduce the impact of friction, the specimen-platen interface was lubricated with vaseline. The sample was compressed until densification occurred, and the compressive stress curve of the porous structure was obtained. For each design, repeatability was determined by examining three samples. This test method has been applied in numerous previous studies [25,26,27,28].

The elastic modulus was derived from the slope of the stress-strain curve in the elastic stage to evaluate the elastic deformation. Yield strength was measured at a strain offset of 0.2%, due to the lack of a distinct peak stress [29]. The formula for energy absorption per unit volume is as follows:

(1) $$W=\int_{0}^{\varepsilon_{0}}\sigma (\varepsilon )d\varepsilon$$

where W is the energy absorption per unit volume, *σ* is the compressive stress, and *ε*_0_ is the compressive strain.

## 3. Results

### 3.1. Theoretical Analysis Models

Ashby classifies the mechanical behavior of porous structures into tensile-dominated and bending-dominated types according to Maxwell’s criterion [30].

In two dimensions, the equivalent equation is [30]:

M = b − 2j + 3(2)

In three dimensions, the equivalent equation is [30]:

M = b − 3j + 6(3)

where b is the number of struts, and j is the number of frictionless joints. When M < 0, it is bending-dominated, and the deformation mechanism is mainly bending deformation; when M ≥ 0, it is tensile-dominated, and the deformation mechanism is mainly tensile or compression deformation.

In the BCC structure, M < 0. Therefore, it is a bending-dominant type, and the deformation of each strut after being loaded is due to resistance to bending.

The area moment of inertia was achieved to evaluate the ability of the section to resist bending; the equivalent equation is

(4) $$I_{x}=\int_{A}y^{2}dA$$

where I_x_ is the area moment of inertia for the x-axis, *y* is the distance to the x-axis, and *A* is the area of the cross-section.

For a tri-directional elliptical cylindrical section (TEC) and a cylindrical section (RD) of equal area, the area moments of inertia for the x-axis are I_xTEC_ and I_xRD_, respectively.

(5) $$I_{xTEC}\;>\;I_{xRD}$$

This indicates that the TEC has a better ability to resist bending. Normal stress can be calculated from

(6) $$\sigma_{max}=\frac{M_{max}}{W_{x}}$$

where M_max_ is the bending moment, and W_x_ is the section modulus.

A study on the lattice structure of 316L stainless steel explored the compression behavior [31]. For BCC-type strut structures with the same cell size, the forces for individual struts are the same for loads of the same [001] direction, so the bending moment is M_TEC_ = M_RD_.

(7) $$W_{xTEC}=\frac{I_{xTEC}}{y_{maxTEC}}$$

(8) $$W_{xRD}=\frac{I_{xRD}}{y_{maxRD}}$$

where W_xTEC_ is the section modulus of the TEC, and W_xRD_ is the section modulus of an RD.

After calculation, W_xTEC_ < W_xRD_, so σ_maxTEC_ > σ_maxRD_, which indicates that the maximum weight that can be carried by a single strut of the TEC is higher than that tolerated by a cylindrical section (RD) in bending-dominated lattice structure.

### 3.2. Compressive Properties

**Figure 6:**
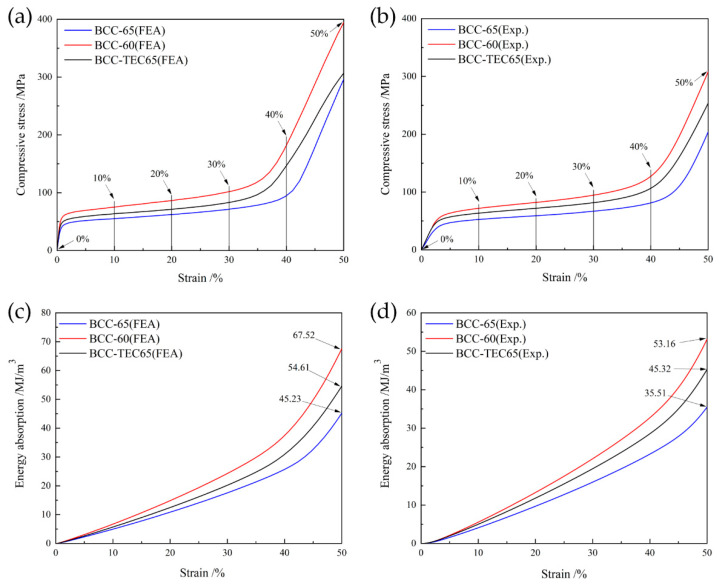
FEA results for quasi-static compression stress-strain curves (**a**) and energy absorption per unit volume curves (**c**); experimental results for quasi-static compression stress-strain curves (**b**); and energy absorption per unit volume curves (**d**).

Figure 6a,b depict the quasi-static compression stress-strain curves of the FEA and experimental results. Like all other lattice structures, their mechanical performance relies on the stretching and bending of the struts [32,33].

The stress-strain curves of all specimens contain the following three stages: the linear elastic stage, plateau stage, and densification stage. In the elastic stage, the stress rises sharply as strain increases within a limited strain range of 5%, but the bending phenomenon of the strut for the lattice structure is not obvious, and the deformation can be recovered. Thereafter, the stress changes a little in the plateau stage with increased strain (almost 40%), and the curve rises in a flat trend where the stress in the plateau stage is flat, which is related to the excellent ductility of 316L. At the end of the plateau stage, many lattice struts come into contact with each other, and densification occurs with the sharply increased stresses.

The strut size, measured porosity, elastic modulus, yield strength, and energy ab-sorption capacity of the specimens for the three typical structures are listed in Table 2. Both BCC-60 and BCC-65 are conventional BCC structures. BCC-60 had a larger strut size (thicker strut) and therefore it had a larger elastic modulus and yield strength than BCC-65.

The porosity of BCC-60 was nearly 5% smaller than that of BCC-TEC65, but the slope of the stress-strain curve at the elastic stage was similar, and the modulus of elasticity was close at about 2120 MPa; thus, it saves nearly 5% of raw material by resisting the same elastic deformation. The yield strength of BCC-60 was greater than that of BCC-TEC65. From this, it can be inferred that there exists a conventional BCC structure with strut sizes ranging from 897.5 μm to 974.3 μm which is capable of yielding the same strength as BCC-TEC65.

BCC-TEC65 had a closed porosity, like BCC-65, but its modulus was greater than that of BCC-65, resulting in a 24.99% increase in the modulus. The yield strength of BCC-TEC65 was larger than that of BCC-65 with an increase of 21.66%. The results for the elastic modulus, yield strength, and energy absorption align with results for other previously reported alternatives, such as cubic and honeycomb structures manufactured from 316L [18,23,24].

The stress-strain curves obtained using the finite element method, as shown in Figure 6a,c and the present experiment, as shown in Figure 6b,d, have a similar trend. The orders of elastic modulus and the yield strength of the three lattice structures obtained using the numerical simulation method are similar to those found in the experimental results, namely:

Elastic modulus (experiment and FEA): BCC-60 ≈ BCC-TEC65 > BCC-65;

Yield strength (experiment and FEA): BCC-60 > BCC-TEC65 > BCC-65.

The experimental compressive strength was greater than the compressive yield strength calculated using the numerical simulation method; however, the difference was less than 18%, consistent with earlier studies [29,34]. This error is mainly due to the fabrication process, and the samples fabricated using YLMs-1 had some errors in the design model in terms of geometry, surface accuracy, and internal defects, such as strut waviness. This case can be used for complex porous structure design [35].

**Figure 7:**
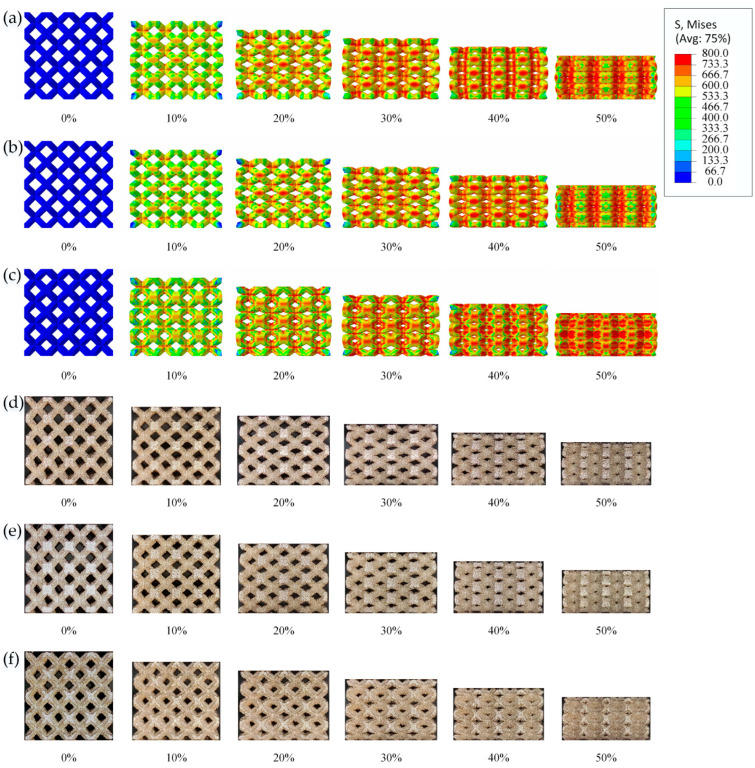
FEA during quasi-static compression of BCC-65 (**a**), BCC-60 (**b**), and BCC-TEC65 (**c**) models; camera frames during quasi-static compression of BCC-65 (**d**), BCC-60 (**e**), and BCC-TEC65 (**f**) models.

Figure 7 shows the typical images captured by the simulation and the camera at different strains during the compression testing. Six images with strain values of 0%, 10%, 20%, 30%, 40%, and 50% were selected for comparison, and it can be observed that the deformation trends of the simulation and experiment are basically the same.

The deformation modes of the lattice structures were homogeneous along the com-pression direction, the stress distributions in the cells of the lattice structures were very similar, and no-slip fracture zones were present in all lattice structures, verifying that the lattice structures were predominantly bending-dominated, which is consistent with the results of a previous study [36].

### 3.3. Energy Absorption Properties

Figure 6c shows the curves of the energy absorption capacity obtained using numerical simulation methods, and Figure 6d shows the energy absorption capacity obtained in the present compressive experiments. The experimental curves of the three lattice structures exhibit the same trend as the curves derived through numerical simulation. The energy absorption capacities of BCC-65, BCC-60, and BCC-TEC65 were 45.23 MJ/m^3^, 67.52 MJ/m^3^, and 54.61 MJ/m^3^, respectively. The energy absorption capacity of BCC-TEC65 was 20.50% higher than that of BCC-65. The experimental results indicate that the BCC-TEC65 has a higher energy absorption capacity.

## 4. Conclusions

In this study, a novel strut was designed for the BCC lattice structure, which consists of tri-directional elliptical cylindrical section (TEC). The novel BCC lattice structure with the TEC strut was prepared via selective laser melting (SLM) of 316L. Scanning electron microscopy revealed that the struts sections were macroscopically “petal-like” shapes and node sections in the [001] direction were macroscopically “4-pointed stars” shapes. The mechanical properties of various lattice structures were simulated using numerical simulations, and it was predicted that a novel lattice structure would have superior mechanical properties. Compression experiments showed that when the porosity was nearly similar, the elastic modulus and compressive yield strength of the novel lattice structure increased by 24.99% and 21.66%, respectively, compared with the conventional lattice structure. In addition, the novel lattice structure had a 20.50% higher energy absorption capacity than the conventional lattice structure. These compression experiment results validate the simulation results acquired using the finite element method. The present design method can be used for targeted parametric design of lattice structures by controlling the shape parameters of elliptic curves, such as the long axis, short axis, and eccentricity, with a greater possibility of diversified parameter control, and can be expanded to other lattice structures, such as BCCZ, FCC, etc., to improve and enrich the lattice structure types, providing good choices for practical applications. Advanced lattice structures have shown promising applications in aerospace, land, and deep-sea fields, such as impact-resistant armored-vehicle door panels, aircraft shells, and submarines for high-pressure environments, due to their good compressive and energy-absorbing capabilities.

## Figures and Tables

**Table 1:** Typical lattice structures.

No.	Lattice Type	Refs.
1	Cubic structure	[6]
2	Quasi-crystalline structure	[7]
3	TPMS structure	[6,8,9,10,11]
4	Plate-strut hybrid structure	[12]
5	Pierced-plate structure	[13]
6	Curved-strut structure	[14]
7	Structural asymmetrical structure	[14]
8	Plate structure	[15]
9	Auxetic structure	[16,17]
10	Other novel structures	[18]

**Table 2:** Geometric dimension and compression properties of the BCC-65, BCC-60, and BCC-TEC65 models.

Lattice Type	Measured Porosity/%	Strut Thickness/μm	Elastic Modulus/MPa	Yield Strength/MPa	Energy Absorption/MJ/m^3^
BCC-65	64.23	d = 897.5 ± 4.6	1588.61 ± 92	34.86 ± 2.03	45.23 ± 4.63
BCC-60	59.31	d = 974.3 ± 7.1	2138.54 ± 86	48.27 ± 1.53	67.52 ± 7.03
BCC-TEC65	63.69	2a = 812.1 ± 4.8, 2b = 351.9 ± 5.1	2117.89 ± 79	42.41 ± 1.64	54.61 ± 6.69

## Data Availability

Not applicable.
